# Granulomatous Mastitis-Related Large Skin Defect in a Patient With Ulcerative Colitis on Mesalazine Therapy

**DOI:** 10.7759/cureus.77498

**Published:** 2025-01-15

**Authors:** Mitsuko Manabe, Shoji Oura

**Affiliations:** 1 Surgery, Kishiwada Tokushukai Hospital, Kishiwada, JPN

**Keywords:** granulomatous mastitis, large skin defect, lattisimus dorsi musculocutaneous flap, mesalazine, ulcerative colitis

## Abstract

A 30-year-old woman with ulcerative colitis on mesalazine therapy developed a breast mass seven months after her first child delivery. Breast massage to the affected breast and drainage of the abscess cavity only resulted in skin necrosis just above the breast mass followed by skin defect formation larger than 10cm in size. A pathological study of the exposed breast tissue led to the diagnosis of granulomatous mastitis (GM). Due to the shrunken but still large skin defect after successful steroid therapy, the patient underwent latissimus dorsi musculocutaneous (LDMC) flap grafting in order to get both better cosmetic outcomes and a shorter treatment period. The patient was discharged without any complications on the eighth day after the operation and has been well with excellent cosmetic outcomes while taking care of her child for 31 months. In conclusion, mesalazine therapy might correlate with this type of large skin defect formation in GM. Breast surgeons should note that LDMC flap grafting can be a feasible therapeutic option to cover large skin defects even in GM patients.

## Introduction

Granulomatous mastitis (GM) is a benign disorder often observed in young women. GM has been postulated to have a variety of causes, including infection [1,2]. In particular, autoimmunity has been pointed out as one of the main GM etiologies because GM highly occurs in women after their first child delivery. Steroid therapy, therefore, has long been the mainstay in the treatment of GM. GM itself is a benign disorder and heals even without any treatment. Spontaneous healing, however, requires a very long time of 5-20 months [3,4] and needs constant wound care which has a major negative impact on the patient’s quality of life.

Infection-induced mastitis forms abscesses close to the skin of and around the nipple-areolar complex due to some kind of bacterial infection through the nipple [5]. In contrast, GM forms abscesses that often extend deep into the mammary gland. However, regardless of etiology, mastitis rarely causes large skin defects to be treated.

Ulcerative colitis (UC) is an intractable disease of unknown cause. Although there are no established methods to completely cure UC, the advent of 5-aminosalicylic acids including mesalazine has made it possible to effectively control unpleasant symptoms of mild to moderate UC.

We herein report a GM patient who developed a large skin defect during mesalazine therapy for UC and was successfully treated with multidisciplinary treatment including latissimus dorsi musculocutaneous (LDMC) flap grafting to the skin defect site [6].

## Case presentation

**Figure 1:**
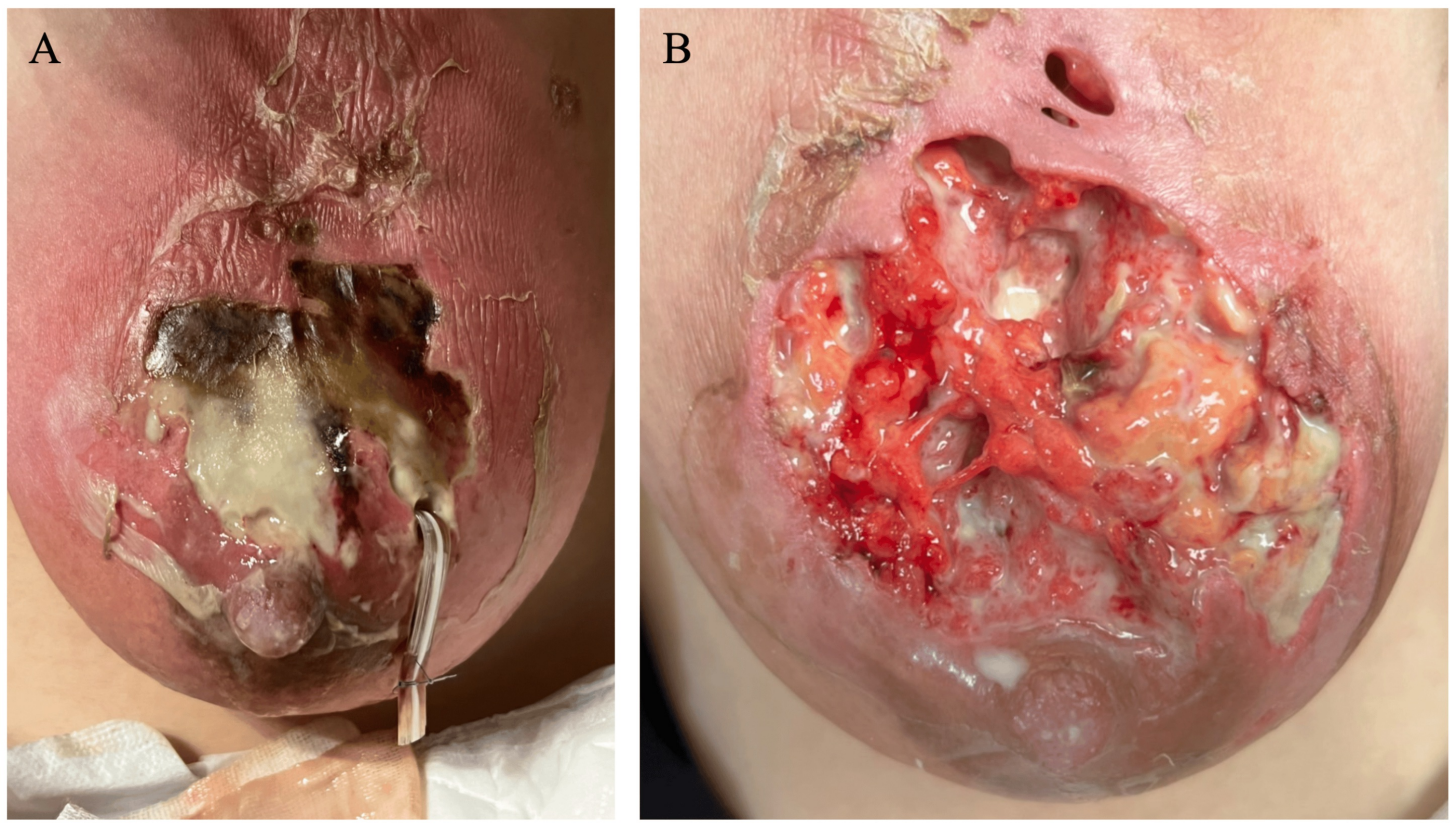
Breast condition A. A photograph taken by her mother showed large skin necrosis. B. A large skin defect and milk retention were observed in the upper part of her left breast.

A 30-year-old woman with well-controlled UC on mesalazine therapy (1200mg×4 for three years) had delivered her first child seven months before. The patient had no history of other diseases including gynecologic disorders. The patient noticed a left breast mass without any flare of UC and consulted the attending obstetrician. Under the tentative diagnosis of galactocele by the doctor, the patient initially received breast massage, unfortunately leading to rapid skin color change (Figure 1A). Drainage of the abscess cavity by the obstetrician further caused skin necrosis and a large skin defect. The patient, therefore, was referred to our hospital for the treatment of the large skin defect. On her first visit to our hospital, a large skin defect, larger than 10cm in size, and milk retention were observed on the exposed mammary gland (Figure 1B).

**Figure 2:**
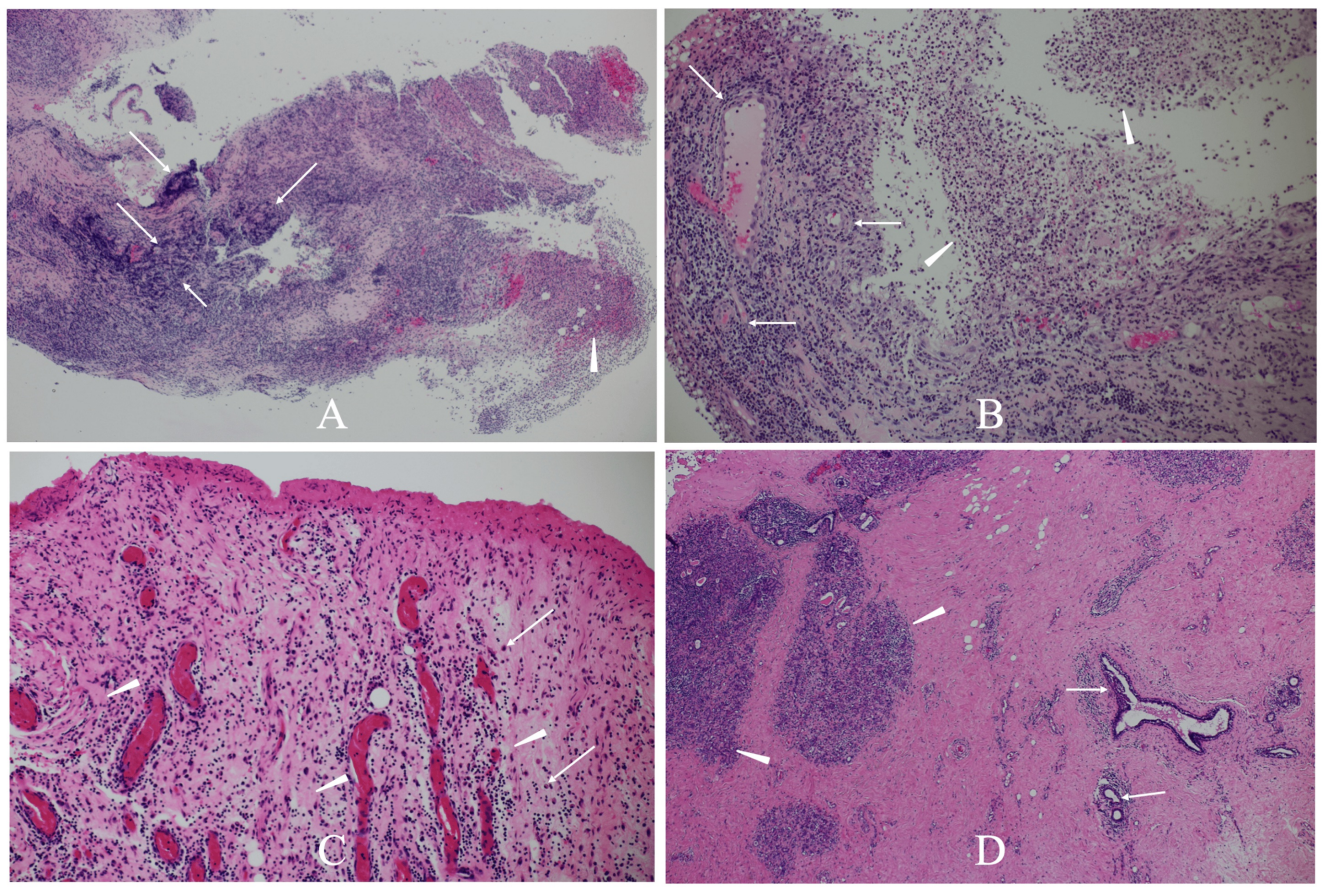
Pathological findings A. Low-magnification view of the exposed breast tissue pathologically showed centrilobular lymphocyte and plasma cell infiltration (arrows) and granuloma formation (arrowheads). B. Magnified view showed numerous lymphocytes around the lobules (arrows) and multiple neutrophils (arrowheads). C. The postoperative pathological study showed fibrosis (arrowheads) and lymphocyte and plasma cell (arrows) infiltration around the vessels. D. The postoperative pathological study showed mild lymphocyte infiltration around the lobules (arrowheads) and mammary ducts (arrows).

Mammary glands could not be assessed with mammography and ultrasonography due to the skin defect. A pathological study using a tiny tissue of the exposed mammary gland showed centrilobular lymphocyte/plasma cell infiltration and granuloma formation with neutrophil infiltration (Figures 2A, 2B), leading to the diagnosis of GM.

**Figure 3:**
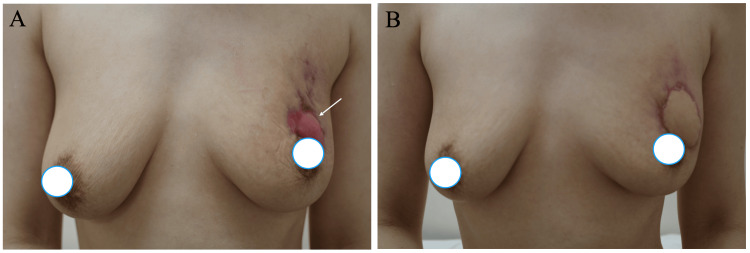
Cosmetic outcomes A. Although the skin defect size significantly decreased, a large skin defect (arrow) was still observed after steroid therapy. B. The large skin defect was successfully covered with the latissimus dorsi musculocutaneous flap without nipple deviation.

The patient, therefore, received steroid therapy and got marked improvement of the GM. The large skin defect significantly shrank but remained still large even after the steroid therapy (Figure 3A). The patient, therefore, underwent LDMC flap grafting to cover the skin defect two weeks after the completion of four-week steroid therapy. The postoperative pathological study of the exposed breast tissue showed massive granulation tissue, slight periductal lymphocyte infiltration, and no active GM findings (Figures 2C, 2D). The patient was discharged without any complications on the eighth day after the operation and has been well with excellent cosmetic outcomes while taking care of her child for 31 months (Figure 3B).

## Discussion

Mesalazine eliminates reactive oxygen species released from inflammatory cells and suppresses inflammation and tissue damage, thereby alleviating the symptoms of UC [7]. Mesalazine is effective when administered topically but is known to migrate into blood from topical sites, although in extremely low concentrations. Mesalazine, therefore, may have some effect on various inflammation including GM. This hypothesis seems to be correct due to the absence of large skin defect reports in GM patients to date [8]. It, however, is naturally unclear what mechanism caused the large skin defect in this case.　

Correlation between GM and autoimmune disorders has been pointed out [2]. The patient, however, did not present with a flare of UC before the onset of GM. We, therefore, judge it highly unlikely that an immune response associated with UC caused the skin defect in this case.

Patients with UC can develop pyoderma gangrenosum [9] and typically develop skin erosion and ulcers, which resemble the skin lesion observed in this case. Pyoderma gangrenosum, however, initially occurs on the skin and extends deeply toward the muscles and bones. This patient, however, initially developed a breast mass followed by skin ulceration. In addition, pyoderma gangrenosum is pathologically classified as neutrophilic dermatosis [10] and is completely different from the pathological findings in this case.

Regardless of the etiology, mastitis often develops fistulas to the skin. The size of a GM skin fistula is essentially the same as that of an infection-induced subareolar abscess fistula. These small skin defects can easily be covered by epithelization once the inflammation subsides. However, large skin defects cannot be cured only by epithelization and need, even if possible, a very long time. For young mothers who have to take care of their babies, these long treatment periods place huge burdens not only on the patients but also on their families. In addition, epithelialization of skin defects is usually accompanied by skin defect size reduction, generally causing the deviation of the nipple-areolar complex to the skin defect direction.

Several therapeutic options can be available for skin defect covering such as free skin grafts, skin substitutes, and musculocutaneous flaps. However, both the steroid use and the possible residual inflammation in the mammary gland, in this case, made the free skin graft and skin substitutes inappropriate for skin defect cover. LDMC flap grafting seemed best for the skin defect cover in GM patients due both to the skin defect location and the stable blood supply to the LDMC flap.

LDMC flap grafting markedly contributed to the successful skin defect cover with minimal in-hospital stays in this case. Short treatment periods are preferable for young mothers who have babies to be cared for. Therefore, in treating GM patients with large skin defects, physicians should note that LDMC flap grafting after the completion of steroid therapy can bring about favorable cosmetic outcomes and shorter treatment periods.

## Conclusions

This is the first case report of a GM-induced large skin defect successfully treated. Physicians should be aware that mesalazine treatment might induce a large skin defect in GM patients. In addition, breast surgeons should note that LDMC flap grafting can be an excellent therapeutic option for covering large skin defects in GM patients.
